# Auxins and Cytokinins—The Role of Subcellular Organization on Homeostasis

**DOI:** 10.3390/ijms19103115

**Published:** 2018-10-11

**Authors:** Vladimír Skalický, Martin Kubeš, Richard Napier, Ondřej Novák

**Affiliations:** 1 Laboratory of Growth Regulators, Centre of the Region Haná for Biotechnological and Agricultural Research, Institute of Experimental Botany of the Czech Academy of Sciences & Faculty of Science of Palacký University, Šlechtitelů 27, 78371 Olomouc, Czech Republic; vladimir.skalicky@upol.cz; 2 Department of Chemical Biology and Genetics, Centre of the Region Haná for Biotechnological and Agricultural Research, Faculty of Science of Palacký University, Šlechtitelů 27, 78371 Olomouc, Czech Republic; martin.kubes@upol.cz; 3 School of Life Sciences, University of Warwick, Coventry CV4 7AL, UK; richard.napier@warwick.ac.uk

**Keywords:** auxin, cytokinin, phytohormone metabolism, phytohormone transport, cellular level, subcellular level

## Abstract

Plant hormones are master regulators of plant growth and development. Better knowledge of their spatial signaling and homeostasis (transport and metabolism) on the lowest structural levels (cellular and subcellular) is therefore crucial to a better understanding of developmental processes in plants. Recent progress in phytohormone analysis at the cellular and subcellular levels has greatly improved the effectiveness of isolation protocols and the sensitivity of analytical methods. This review is mainly focused on homeostasis of two plant hormone groups, auxins and cytokinins. It will summarize and discuss their tissue- and cell-type specific distributions at the cellular and subcellular levels.

## 1. Introduction

**Figure A1:**
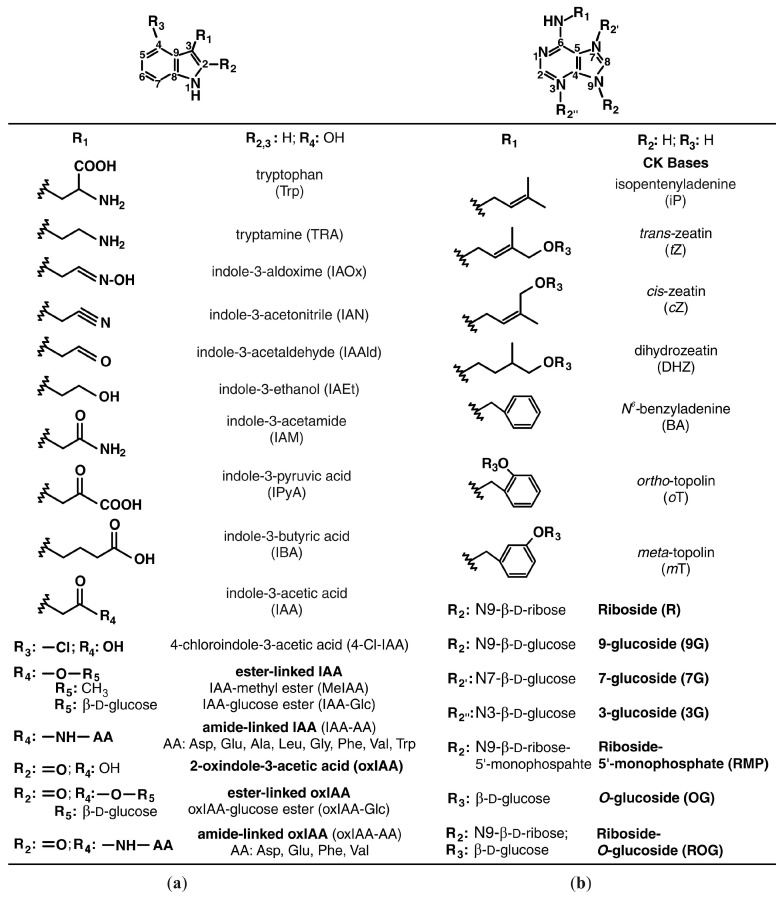
Structures, names, and abbreviations of naturally occurring (**a**) auxins and (**b**) CKs. Wavy cuts indicate position of substituent attachment.

The most well-documented groups of plant hormones are auxins and cytokinins (CKs) (Figure A1) with reasonably well-described signaling, transport, and metabolism (biosynthesis, conjugation, and degradation). Moreover, mutual auxin–cytokinin regulation and/or crosstalk appear to control many developmental processes in plants [1]. Since the 1950s, both CKs and auxins have been known for their ability to effectively determine the type of organs regenerated in vitro from undifferentiated callus cultures [2]. High auxin-to-CK ratios stimulate root formation, whereas low ratios promote shoot formation. Müller and Sheen [3] showed that antagonism between CK and auxin is primarily realized at the molecular level and is important for specifying root stem cells during early embryogenesis. Moreover, recent transcriptomic data have shown that meristems reform in positions determined by antagonistic auxin and CK signaling domains during tissue repair [4]. On the other hand, synergistic effects of auxins and CKs have also been reported, an example being shoot apical meristem formation [5,6].

The importance of phytohormone homeostasis at the cellular level has become more prominent with the increasing sensitivity of analytical tools [7]. It is generally accepted that compartmentation is a key feature of eukaryotic cells. Plant cells contain admirably complex, albeit well-organized membrane systems dividing them into organelles or compartments. This partition provides possibilities to create appropriate microenvironments and conditions for specialized metabolic pathways. Thus, unique sets of enzymes, transporters, and other proteins are found separated into organelles.

Current advances in indirect or direct visualization methods and other sensitive analytical techniques enable us to visualize phytohormone distributions in vivo at the cellular and subcellular levels. In this review, the authors have connected homeostasis (transport and metabolism) of auxins and CKs with their tissue- and cell-type specific distributions at the cellular and subcellular levels. They are convinced that this topic will open completely new horizons in understanding how the balance of plant hormones is created and controlled.

## 2. Organelle-Specific Phytohormone Profiling

Analytical methods for quantitation of auxins and CKs have become increasingly sensitive and capable of discriminating not only the free hormones, but also many of their precursors, metabolites, and catabolites [7,8]. Nevertheless, at the subcellular level, organelle-specific phytohormone profiling is challenging and many factors need to be optimized, such as (i) leakage during isolation; (ii) purity of isolated compartments; and (iii) dynamic metabolic changes during isolation.

### 2.1. Subcellular Fractionation

The original idea of separating intracellular compartments to study the partition of enzyme processes was developed by De Duve and co-workers in the 1950s [9]. Methods of organelle isolation are mainly based on differential centrifugation or density gradient ultracentrifugation [10,11,12,13,14]. Even the simplest differential centrifugation can provide enriched fractions of crude organelles [15,16]. Higher purity organelle fractions can be achieved by density gradient ultracentrifugation yielding fractions enriched in endoplasmic reticulum (ER) [14], Golgi apparatus [17], vacuoles [11], mitochondria [16], and chloroplasts [12,18].

Alternative methods for compartment separation have been also described, for example, two-phase partitioning [19] and non-aqueous or aqueous fractionation [20,21]. Techniques such as flow cytometry can be used for more rapid sorting of organelles labelled by fluorescent probes, for example, nuclei [22], chloroplasts [23], and mitochondria [24]. Affinity capture or pull down by magnetic microparticles has been also used for isolating nuclei [25] or mitochondria [26].

### 2.2. Phytohormone Profiling in Organelles

**Figure 1:**
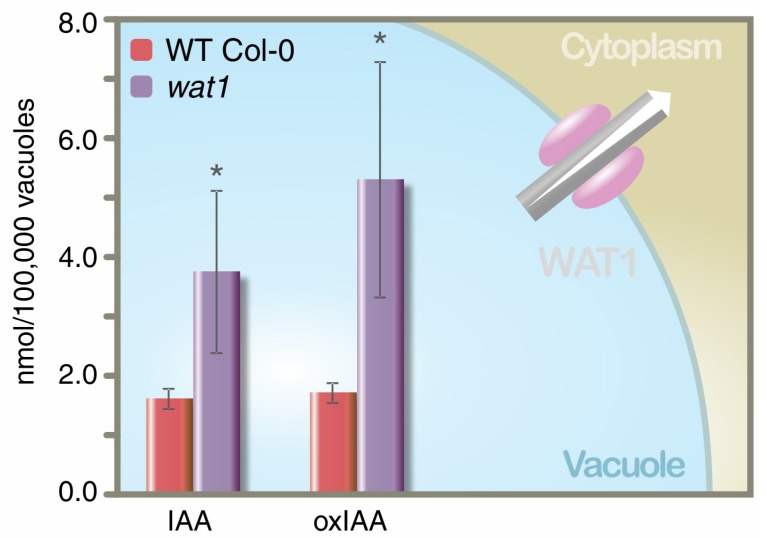
Indole-3-acetic acid (IAA) and 2-oxindole-3-acetic acid (oxIAA) contents were measured in vacuolar fractions isolated by density gradient ultracentrifugation from the wild-type (*Arabidopsis* Col-0) and the vacuolar auxin transporter mutant line *(wat1-1*). Plant tissues were grown, and vacuole isolation was performed as previously described [31]. Samples were purified by in-tip solid-phase microextraction [32] using a minor modification of the protocol described by Pěnčík et al. [33]. Quantification of IAA and oxIAA was performed by LC-MS/MS [34]. The bars represent averages (±SD) of four independent biological replicates; the asterisk indicates *p*-values of the genotype comparisons in an ANOVA analysis (* *p* < 0.05). White arrow indicates flux direction.

Currently, there are only few reports dealing with the determination of phytohormones in live-cell systems [7]. In addition, little is known about extra- and intracellular phytohormone distribution, or the phytohormone levels in individual cell compartments. Indole-3-acetic acid (IAA) has been determined in chloroplasts and mitochondria [27], whereas CKs, IAA, and abscisic acid concentrations have been determined in chloroplasts [28,29] (Table 1). The full profile of IAA and its metabolites has been described in wild-type *Arabidopsis thaliana* vacuoles [30] with determinations providing, for example, clear functional evidence of the vacuolar auxin transport protein WALLS ARE THIN 1 (WAT1) (Figure 1).

Profiling of CK metabolites at the subcellular level has been performed in both *Arabidopsis* and barley (*Hordeum vulgare*) [31]. Concentrations of 25 CK metabolites were determined from isolated apoplast, cytosol, and vacuoles (Table 1). Surprisingly, the highest proportion of CKs was located outside the cell (up to 90%, with a majority as *O*- and *N*-glucosides), and only about 10% was present in cytosol and vacuoles. In transgenic barley expressing the cytokinin oxidase gene *AtCKX1*, severe decreases in extracellular *trans*-zeatin (*t*Z) and *t*Z-7-glucoside (*t*Z7G) were accompanied by compensatory increases of isopentenyladenine (iP) and vacuolar isopentenyladenosine (iPR).

All these practical examples indicate that a far richer picture can be drawn of phytohormone homeostasis and fluxes with higher resolution data, but hormone profiling and quantitation remain challenging at the resolution required for reliable data about subcellular compartmentation.

## 3. Auxins

**Figure 2:**
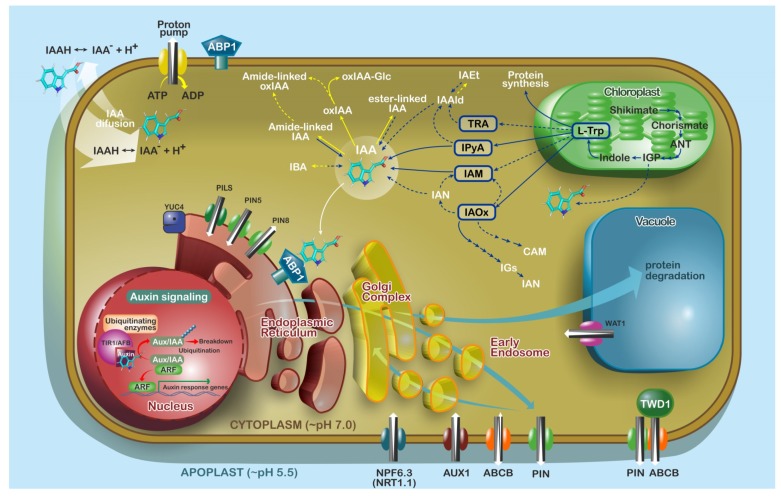
Model of cellular and subcellular auxin homeostasis and signaling in *Arabidopsis*. IAA biosynthesis (indicated by dark blue arrows) could be mediated by l-tryptophan (l-Trp)-dependent or independent biosynthetic pathways [47]. Tryptophan as a substrate for the synthesis of IAA is synthesized in stroma of chloroplast [40]. There are already four described biosynthetic pathways named according to their first intermediates (in dark blue rectangles [60]). In *Arabidopsis*, IAA biosynthesis is running predominantly via the indole-3-pyruvic acid (IPyA) pathway including: cytoplasmic TRYPTOPHAN AMIDOTRANSFERASE OF ARABIDOPSIS (TAA1), TAA-Related (TAR1) localized on plasma membrane (PM), and YUCCA4 attached to the ER membrane [47,52]. Free IAA levels can be modulated via conjugation and/or oxidation, rarely via methylation (metabolic pathways are represented by yellow arrows [59,61]. Four main families of active auxin transporters are described: PM localized AUXIN1/LIKE-AUX1 (AUX1/LAX) auxin influx facilitators, and perhaps also into the ER [62]; PINs efflux carriers [36,63]; ATP-binding cassette type B (ABCB) proteins [64] involved in the influx or efflux of auxin [65,66]; and finally PIN-like (PILS) together with short PIN-FORMED proteins (PINs) (PIN5, 6, and 8) with confirmed localization at ER [67]. WALLS ARE THIN 1 (WAT1) is a recently described tonoplast-localized auxin transporter [30]. Similarly, NPF6.3 (NRT1.1) can control auxin influx (transport is marked by white arrows [68,69]. Nuclear TRANSPORT INHIBITOR RESPONSE1/AUXIN SIGNALING F-BOX proteins (TIR1/AFBs) are considered as proven auxin receptors. However, the strong proof that putative AUXIN BINDING PROTEIN 1 (ABP1) and S-PHASE KINASE-ASSOCIATED PROTEIN 2A (SKP2A) receptors directly mediate auxin signaling still remains contentious (signaling is highlighted by red arrows [37,70]). Light blue arrows indicate protein trafficking. Solid arrows indicate known and well-described pathways, dashed arrows indicate not well-defined pathways. Abbreviations and structures of all IAA metabolites (precursors, catabolites, and conjugates) are listed in Figure A1a.

It is well described that cellular IAA concentrations are strictly regulated by its transport, biosynthesis, and catabolism [35]. Changes in auxin concentrations and morphogenic gradients are created in plant tissues and organs as a response to both exogenous and endogenous stimuli, resulting in various developmental events, but how homeostasis is managed in these systems is far from clear. While TRANSPORT INHIBITOR RESPONSE1/AUXIN SIGNALING F-BOX proteins (TIR1/AFBs) are considered as proven auxin receptors, the clear contribution of AUXIN BINDING PROTEIN 1 (ABP1) and S-PHASE KINASE-ASSOCIATED PROTEIN 2A (SKP2A)-dependent perception to auxin signaling still remains controversial [36,37] (Figure 2).

Polar auxin transport (PAT) is a regulated cell-to-cell transport of auxin that provides essential directional and positional information for all vital plant developmental processes, such as vascular differentiation, apical dominance, patterning, organ polarity, embryogenesis, organogenesis, phyllotaxis, and tropisms [38]. Disruption of such directional auxin movement by genetic or pharmacological manipulations results in severe developmental defects [39]. Local auxin production, frequently together with auxin transport, influences lateral root development, embryogenesis, and leaf and fruit development, whereas a strong reduction in auxin levels leads to defects in gravitropism, vasculature development, and reduced apical dominance [40]. All these activities are described at the tissue level, but little is known about how homeostasis, and perturbations to homeostasis, are affected at the subcellular level.

### 3.1. Locations of Auxin Biosynthesis and Metabolism

The first organelle-specific activity connected with auxin homeostasis described indole-3-butyric acid (IBA) enzymatic conversion to IAA in a peroxisome-dependent reaction [41]. However, whereas IBA has been recorded from a number of plant species [35], other labs have had difficulties detecting IBA or report it at much lower concentrations than IAA [34,42]. Certainly, IBA is a poor ligand for the receptor TIR1 [43,44], but IBA and/or its conjugates might still contribute to IAA homeostasis [45].

The biosynthesis of IAA as a natural auxin could be mediated by two main directions: via an l-tryptophan (l-Trp)-dependent or an l-Trp-independent pathway [46,47] (Figure 2). De novo synthesis through the l-Trp-independent pathway is well described in microorganisms [48] but still discussed in higher plants [49,50]. In contrast, l-Trp-dependent pathways are a significant source of endogenous IAA for higher plants [40], as l-Trp is synthesized by the shikimate pathway localized in the chloroplast stroma. Downstream, IAA biosynthesis is predominantly via the indole-3-pyruvic acid (IPyA) pathway with three main family proteins (Figure 2): TRYPTOPHAN AMIDOTRANSFERASE OF ARABIDOPSIS (TAA1 localized in cytoplasm) and TAA-Related (TAR1 localized on plasma membrane (PM)) that are responsible for the synthesis of IPyA from tryptophan, and flavin monooxygenases from the YUCCA family that are responsible for the conversion of IPyA to IAA [51,52] (Figure 2). The YUCCA enzymes are likely to be cytoplasmic, although *Arabidopsis* YUCCA4 can be localized both to the cytosol and to the cytosolic face of the ER membrane [18]. At least three of the maize auxin biosynthetic proteins are also localized to ER membranes [53] (Figure 2).

The indole-3-acetaldoxime (IAOx) pathway is a unique biosynthetic pathway in *Brassicaceae* with cytochrome P450 enzymes CYP79B2 and CYP79B3 localized in chloroplasts, where their substrate Trp is synthesized [54], converting Trp to IAOx, and then to indole-3-acetamide (IAM) or indole-3-acetonitrile (IAN) downstream. However, the enzymatic steps between IAOx and IAN have yet to be identified. The synthesis of IAM from IAOx has been directly demonstrated in assays with *cyp79b2 cyp79b3* mutants [55,56], and IAM hydrolases have been isolated from *Arabidopsis* and tobacco BY-2 cells (AtAMI1 and NtAMI1) and shown to convert IAM to IAA in vitro, but the subcellular localization of these enzymes remains unclear [57,58], despite some evidence of AtAMI1-green fluorescent protein (GFP) fusion protein in the cytoplasm [59].

Free IAA levels are probably managed by activities in the cytoplasm, the compartment of synthesis and of arrival by transport. In the cytoplasm, IAA can be modulated via conjugation and/or oxidation, and rarely via methylation [59]. IAA can be conjugated via ester linkages to glucose by UDP-glucosyl transferases UGT74D1 and UGT84B1 to create 1-*O*-indole-3-acetyl-*β*-d-glucose (IAA-Glc) [71], or to amino acids by the GRETCHEN HAGEN 3 (GH3) family of IAA-amido synthases [72,73]. Interestingly, Barbez and Kleine-Vehn [74] later hypothesized that the localization of the GH3 family is in the ER, but this would place them in the same compartment as ILR1-like amidohydrolases (ILR1, ILR2, and ILR3 [75]), which will hydrolyze the products of GH3s. Unfortunately, clear evidence for GH3 localization to the ER is still missing. If IAA conjugates are synthesized in the cytoplasm, the authors can hypothesize that such conjugates are rapidly transported out of the cytoplasm for storage or derivative pathways.

The GH3 enzymes are induced strongly by elevated concentrations of auxin and this provides one level of homeostatic control [76,77], but the role of the ILRs in subsequently releasing free IAA back from amido-conjugates is not known. The steady state concentration of IAA in the cytoplasm is considered to be 5 μM when calculated in system models [78]. Rises in concentration above the steady state in the cytoplasm and nucleus will induce transcription and translation of *GH3s*, among many other genes, and the analysis above suggests that these reside in the cytoplasm ready to react with the elevated free IAA. Consideration of the kinetic properties of these enzymes suggests that GH3s will become increasingly active in the micromolar range of IAA concentrations (K_m_ for OsGH3-8 = 182 μM, [77]; K_m_ for AtGH3-5 = 700 μM, [79]). While these K_m_ values suggest poor activity at the concentrations of IAA likely to be encountered in the cytoplasm, the enzymes do have very high catalytic efficiencies (k_cat_/K_m_) and so free IAA will be rapidly conjugated by resting levels of enzyme, and this will be rapidly supplemented as new enzyme is generated by the auxin response. In the ER, ILRs will become active at somewhat lower concentrations of the conjugates (K_m_ AtILR1 = 14 μM; [75]), but the proper location of the conjugates remains to be determined.

IAA oxidation to 2-oxindole-3-acetic acid (oxIAA) is the major IAA catabolic pathway in *Arabidopsis* [34,80,81]. It was later shown that another oxidative metabolite in *Arabidopsis*, oxIAA-glucose (oxIAA-Glc), was synthesized via glycosylation of oxIAA and not via oxidation of IAA-Glc [82,83]. Oxidation of some IAA amides in *Arabidopsis* was also detected [80,84]. The first characterized IAA oxidases, DIOXYGENASE FOR AUXIN OXIDATION (DAO) in dicots, were rice OsDAO homologs in *Arabidopsis* AtDAO1 and AtDAO2 [83,85,86]. These dioxygenases are cytoplasmic [83] and so, again, responses to elevations of IAA concentration are targeted to the cytoplasm and one may expect the cytoplasmic concentration at homeostasis to be micromolar or lower given that OsDAO1 actively oxidized IAA when 1 μM IAA was supplied [85].

AtDAO1 was shown to be a primary determinant of auxin homeostasis [83]. However, the work on oxidases [83,86] showed that the loss of IAA oxidation in *atdao1* mutants did not lead to a significant change in IAA levels, suggesting redundancy in homeostatic mechanisms. Moreover, the mathematical model from Mellor et al. [87] suggests that, in *atdao1* mutant, IAA-aspartate (IAA-Asp) and IAA-glutamate (IAA-Glu) accumulate, compensating for the loss of IAA oxidation.

There are several reports indicating that methylation of IAA is highly relevant for some plant developmental processes, such as leaf development [88] and differential growth in the hypocotyl [89].

Taken together, these results suggest that plants possess redundant and sensitive mechanisms to catabolize cytoplasmic IAA [90]. It will be useful in future to know into which compartment the oxidation and other catabolic products are moved. The presence of the amidohydrolases in the ER suggests that this compartment is important, but it remains possible that this is only involved in feedback control of cytoplasmic IAA concentrations.

### 3.2. Auxin Transport

There are four main families of active auxin-specific transporters and by their nature, each is localized to specific membranes (Figure 2). Therefore, one can surmise their roles in auxin homeostasis in some detail: (i) AUXIN1/LIKE-AUX1 (AUX1/LAX) auxin-H^+^ symporters, responsible for auxin transport from the apoplast into the cell, and perhaps also into the ER [62,91,92]; (ii) PIN-FORMED proteins (PINs) that are gradient-driven secondary transporters (efflux carriers) [63]; (iii) ATP-binding cassette type B proteins (ABCBs) [64] uniformly localized at the PM are involved in the ATP-driven influx or efflux of auxin [65,66]; and (iv) the PIN-like (PILS) protein family with confirmed localization at ER [67] (Figure 2). Additionally, it has been demonstrated that the nitrate transceptor NPF6.3 (NRT1.1, Figure 2), which belongs to the NPF (NRT1/PTR) family in *Arabidopsis* [68,69], is involved in the auxin influx in heterologous systems of *Xenopus* oocytes, yeast, and tobacco BY-2 cells [93,94,95]. Finally, the tonoplast-localized auxin transporter WAT1 and endomembrane ADP1, that are involved in maintaining the intracellular auxin homeostasis, were also identified [30,96]. Experiments showed that WAT1 confers auxin efflux to yeast cells and *Xenopus* oocytes [30]. However, it is still not known which auxin-related compound(s) are transported in planta.

In *Arabidopsis*, the PIN family consists of eight members and divides into two subfamilies according to the length of a hydrophilic loop located in the middle of their polypeptide chain. The “long” canonical PINs (PIN1-4, and 7) [97,98,99] act as auxin efflux carriers and are polarly localized at the PM where they direct auxin flow [100,101]. The “short” non-canonical PINs (PIN5-6 and PIN8) have the hydrophilic loop, either partially (PIN6) or significantly reduced (PIN5 and PIN8) [99]. “Short” PINs are predominantly localized to the ER where they presumably regulate auxin homeostasis by pumping auxin into (PIN5) or out (PIN8) of the ER lumen or hypothetically from the ER lumen into the nucleus (PIN6 and PIN8) [14,102,103,104,105]. However, Ganguly et al. [106,107] and Simon et al. [108] revealed dual localizations of PIN5, PIN6, and PIN8 at the PM and ER in *Arabidopsis* epidermal and root hair cells, as well as in tobacco BY-2 cells. PIN5::GFP was predominantly localized to the ER and PIN8::GFP, to the PM. However, in the epidermal and cortical cells of the root meristem region (the PIN2 domain), PIN5 showed a PM localization pattern [106,107]. Finally, Ganguly et al. [107] came up with the hypothesis that both PIN5 and PIN8, with their dual localization property, may act as linkers between the ER-based PILs and the PM-based canonical PINs. It is also clear that PINs do not stay static but undergo constitutive cycling through the clathrin-coated vesicle machinery between the PM and ER compartments [109,110].

ABCB, ABCD, and ABCG protein subfamilies are directly or indirectly involved in auxin transport. There is a clear and well-described functional interaction between members of the ABCB family (ABCB1 and ABCB19) and TWISTED DWARF1 (TWD1) which acts as a chaperone during PM trafficking [111]. Dudler and Hertig [112] were the first to determine the substrate specificity of ABCB1 and later Sidler et al. [113] pointed out the role of ABCB1 in the regulation of hypocotyl elongation and its localization to the PM. ABCB1 and ABCB19 show mainly apolar cellular localizations, although partial apical localization is found in different tissues [114,115,116]. Interestingly, PAT in *abcb19* was highly reduced, in both inflorescence stems and hypocotyls [117], and by ~70% in the *abcb1 abcb19* double mutant, whereas *pin1* exhibited only a ~30% reduction [118,119]. Similar drastic reductions in PAT were found in *twd1* [120]. This suggests that ABCBs primarily contribute to long-distance auxin transport and do not function in establishing the basal auxin flows that regulate organogenesis [111,118,119,121]. It has also been demonstrated that ABCB4 works as a unique auxin concentration-dependent switchable influx/efflux transporter [65,66], and this will clearly contribute to homeostatic control of cytoplasmic auxin concentrations.

The auxin carriers that are specifically localized to ER provide a clear link between auxin compartmentalization and auxin conjugation-based metabolism. Moreover, the role of auxin intracellular transport (PIN5, PIN8 and PILS) together with compartmentalization of auxin metabolism can be interfaced in maintaining and regulating intracellular auxin homeostasis [74]. Mravec et al. [102] were the first to realize that PIN5 increases cellular auxin retention in *Arabidopsis* protoplasts presumably via auxin transport from the cytosol into the ER lumen. Moreover, PIN5 activity decreases cellular levels of free IAA and increases levels of some auxin conjugates, namely, IAA-Asp, IAA-Glu, and IAA-Glc, suggesting a possible role for PIN5 in compartmentalized auxin metabolism. However, the picture for PIN8 is less clear [14,65,66,74,103]. As for PIN5, PILS2 and PILS5 in the ER increase cellular auxin accumulation, but reduce nuclear auxin signaling, and so one can speculate that they promote the sequestration of cytosolic auxin into the ER, where it is unavailable for nuclear auxin signaling [67]. 

It is clear that local directed transport activities contribute significantly to the regulation of cellular auxin metabolism. Indeed, Middleton et al. [122] have combined mathematical modelling with time course data from both auxin-mediated nuclear signaling and quantitative phenotyping at the single cell level, to show that an ER-to-nucleus auxin flux represents a major subcellular pathway to directly control nuclear auxin levels. Based on the preceding, the authors can propose that auxin-mediated responses are controlled by both maintenance of a homeostatic auxin pool in the ER together with regulated rapid auxin fluxes between ER and nucleus.

## 4. Cytokinins

**Figure 3:**
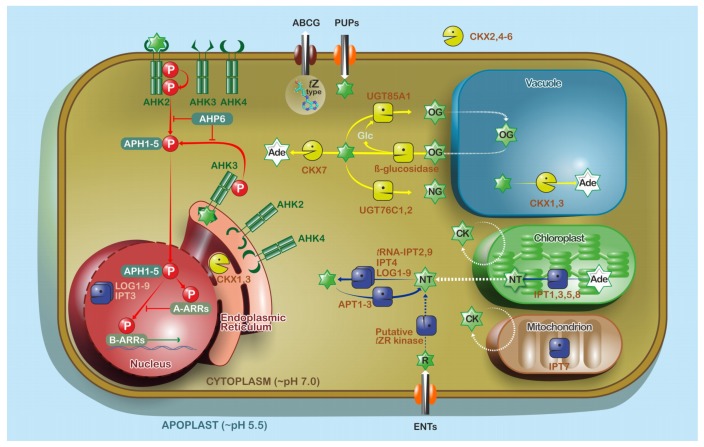
Model of cellular and subcellular CK homeostasis and signaling in *Arabidopsis*. De novo synthesis of CKs is mediated by isopentenyl transferases (IPTs) mainly in chloroplasts; nevertheless, they are localized also in mitochondria, cytosol, and nuclei [137,138]. LONELY GUY enzymes (LOGs) present in cytosol and nuclei are other enzymes, which transform CK nucleotides to active form [140] (biosynthesis is highlighted by dark blue arrows). In contrast, APTs catalyze the opposite reaction [145,146,147]. Most of the active CKs can be modulated by uridine diphosphate glycosyltransferases (UGTs) [148] or *β*-glucosidase [144,165] (yellow arrows mark reversible/irreversible inactivation). The terminal degradation product of cytokinin dehydrogenases/oxidases (CKXs) is adenine (Ade, yellow arrows also mark CK degradation). CKXs are prevalent in the apoplast, although three homologs are intracellular [151,153,154]. Transport (represented by white arrows) of CK free bases and their ribosides to cytoplasm is facilitated by purine permeases (PUPs) [161,166,167] and equilibrative nucleoside transporters (ENTs) [158,168], respectively. Lomin et al. [169] proposed a model where ENTs are involved in *t*ZR transport to cytosol and its subsequent conversion via a putative kinase and LOG into an active CK base, which enters to ER and triggers signaling. ABCG14 was described and proven as an exporter of *t*Z-types [163,164]. CK signaling pathways (marked by red arrows) are initiated by three ARABIDOPSIS HISTIDINE KINASES (AHKs) localized at PM [170] or ER [13,171]. Signal is transmitted via ARABIDOPSIS HISTIDINE PHOSPHOTRANSFER1-5 (AHP1-5) [172,173] to nuclear type-A ARABIDOPSIS RESPONSE REGULATORS (A-ARRs) or B-ARRs (type-B). AHP6 is inhibitory [174,175]. Activation of B-ARRs leads to transcription [176,177] of CK inducible genes including *A-ARRs* which mediate a negative feedback loop [172,178,179,180]. Green stars indicate CK species; Glc—glucose; NT—cytokinin nucleotides; NG—cytokinin *N*-glucosides; OG—cytokinin *O*-glucosides; P—phosphate moiety; R—cytokinin riboside. Solid arrows indicate known and well-described pathways, dashed arrows indicate not well-defined pathways.

CKs are divided according to the chemical character of their side chain on the prevalent isoprenoid group, such as *t*Z, *cis*-zeatin (*c*Z), dihydrozeatin (DHZ), and iP. The same classification applies to benzyladenine (BA) and topolins, which occur less in nature, which are CKs carrying an aromatic group instead of the isoprenoid (Figure A1b) [123,124,125,126,127]. Another group of naturally occurring CKs are derivatives modified at position C^2^ by the methylthio group [128]. CKs promote many responses at the cellular level (e.g., cell cycle and division [129], chloroplast development [130]) and are modulators of PIN formation and polarity [131]. These processes are dependent on CK perception mediated by three ARABIDOPSIS HISTIDINE KINASES (AHKs) which trigger a multistep phosphorelay cascade leading to gene transcription. The AHK receptors sit mainly in the ER, but the PM might be relevant in some circumstances as well (described in detail in [132,133]) (Figure 3).

### 4.1. Locations of Cytokinin Biosynthesis and Metabolism

Key enzymes catalyzing the first step of CK biosynthesis are the isopentenyl transferases (IPTs). IPTs mediate the conjugation of an isopentenyl group to the *N*^6^-position of the adenine ribotide to form isopentenyladenosine-5′-di- or -triphosphate (iPRDP or iPRTP, respectively). Several IPTs were identified in *Arabidopsis* (AtIPT1-9) [134,135,136], where AtIPT2 and 9 can also catalyze isopentenylation of tRNA to provide a source for *c*Z-type CKs [136]. AtIPT1, 3, and 5 fused with GFP were localized to the chloroplasts of mesophyll cells [137], although AtIPT3 appears also in the nucleus. Specific localization depends on posttranslational modifications such as farnesylation, which may overcome the presence of chloroplast transit peptides [138] (Figure 3). GFP fusions of AtIPT2 and AtIPT4 point to cytosolic localization. This finding agrees with the idea that AtIPT4 may utilize isoprenoid precursors synthetized via the mevalonate pathway in the cytosol, but it is likely that the main pool of *t*Z arises from plastids. Additionally, AtIPT7::GFP was observed in mitochondria [137] (Figure 3).

Synthesis of iPRDP and iPRTP nucleotides via transmission of isoprenoid moieties to adenosine is followed by hydroxylation to produce *t*Z-type CKs, a reaction catalyzed by cytochrome P450 monooxygenases CYP735A1 and CYP735A2 [139]. CK nucleotides can get phosphoribohydrolased by “LONELY GUY” (LOG) enzymes into highly active free-base forms [140]. To date, nine AtLOG homologs targeted predominantly to the nucleus and cytosol have been identified [141].

It is expected that the metabolism of active free bases at least partially regulates CK homeostasis. CK bases can be reversibly conjugated with sugars (e.g., glucose or xylose) through their hydroxyl moiety on the *N*^6^-side chain of *t*Z, *c*Z, and DHZ via *Arabidopsis* uridine diphosphate glycosyltrasnferase (AtUGT) 85A1 which is located to the cytosol [142,143] (Figure 3). A pool of *O*-glucosides (OG) could serve as CK storage with the potential of rapid conversion back to active CKs via *β*-glucosidases [144]. Another reversible inactivation could be mediated by enzymes common with purine metabolism, such as adenine phosphoribosyltransferases (AtAPT1-3), which appear to be cytosolic and act antagonistically to other AtLOGs (Figure 3), switching bioactive CKs back to nucleotides [145,146,147]. Direct glycosylation at N7 or N9 might also be catalyzed by cytosolic UGT76C1 or UGT76C2, causing irreversible CK inactivation [148].

Cytokinin dehydrogenases/oxidases (CKXs) are recognized as the main enzymes mediating CK degradation [149,150] and they play a key role in the maintenance of endogenous CK levels. The seven CKX homologs in *Arabidopsis* have distinct subcellular localizations. It seems that the main site of CK inactivation is localized in the apoplast by CKX2 and CKX4-6 [151]. CKX1 and 3 were initially predicted as mitochondrial based on in silico experiments [152], although later GFP fusions showed that these two enzymes are predominantly targeted to vacuole, with some observed signal also in ER [151] where they are catalytically active [153]. In the case of CKX7, the lack of a signal peptide suggests that it is localized to the cytosol [154] (Figure 3).

As well as localizations, AtCKXs differ in their substrate specificity adding a level of complexity to cytokinin homeostasis above that for auxin. While CKXs prefer unsaturated isoprenoids, aromatic CKs can also be degraded but with lower turnover rates [155,156] and DHZ, OG, and almost all *c*Z-types are believed to be resistant to AtCKXs.

### 4.2. Cytokinin Transport

CKs are long-distance signals and the different CK forms appear to be moved differentially. For example, *t*Z riboside (*t*ZR) is transported acropetaly in xylem sap, whereas iPR is mainly transferred basipetaly via phloem [157,158,159,160]. Despite the importance of CK transport, the facilitator proteins were not discovered until the beginning of the 21st century when three protein groups possessing CK translocation activity were described: purine permeases (PUPs) [161], equilibrative nucleoside transporters (ENTs) [162], and the G subfamily of ATP-binding cassette (ABCG) transporters [163,164] (Figure 3). CK transport seems to be shared with essential nucleobases, although the molecular basis of CK transport is still poorly understood compared with auxin transport.

The PUP family numbers 23 members [132,161] and some PUPs appear to mediate CK uptake at the PM (Figure 3). AtPUP1 was first examined in a yeast mutant deficient in adenine uptake. Results suggested that kinetin and *t*Z, but not *t*ZR, were substrates by competitively inhibiting adenine uptake. A mildly acidic apoplast raised AtPUP1 activity, whereas proton pump inhibitors reduced it. These findings point to energy-dependent and potentially proton-coupled transport against the concentration gradient [161], later confirmed by the transport of radiolabeled *t*Z [166]. However, PUPs are promiscuous to other purines and even though PUP14, for example, was shown to be involved in the early stages of plant development [167], their role specifically as CK carriers remains to be elucidated.

AtENT1 was described as a putative nucleoside transporter based on shared similarity with human ENTs [181] and proton-dependent import was confirmed later [162,182]. AtENT1-8 are localized at the PM [162,181,183] (Figure 3), although AtENT1 was also identified in the tonoplast proteome [184]. Substrate specificity of some *Arabidopsis* ENTs has been examined using competition assays of adenosine uptake. As a result, AtENT6 and 8 may participate in CK riboside transport and AtENT6 preferred iPR to *t*ZR [158,168].

ABCG14 was revealed as the first described CK exporter involved in root-to-shoot transport of CKs. It is highly expressed in *Arabidopsis* root vascular tissue and loss-of function abcg14 mutants resemble CK-deficient phenotypes [163,164], and measurements showed that mutant shoots contained decreased *t*Z-type CKs, despite abundant *t*Zs in roots. Interestingly, iP-type and *c*Z-type CK contents were elevated in both shoots and roots, suggesting that the *abcg14* plants are attempting to compensate for the loss of transport of root-synthetized *t*Z-type CKs for intrinsic CK homeostasis [163,164]. Undoubtedly, AtABCG14 represents an important element in the long-distance transport of CKs. Unfortunately, there is little information on local or subcellular compartmentation of cytokinins or cytokinin catabolites.

## 5. Future Perspectives

In spite of many recent studies on plant hormones, there are still gaps in our knowledge about the mechanisms of homeostasis. For instance, detailed information about intracellular CK transport is still missing. Cell- and organelle-specific distributions of auxin, CK, and their related compounds are also waiting for elucidation. Auxin and CK profiling at the subcellular level will definitely open new insights and provide a better understanding of the regulation of auxin and CK homeostasis, offering more precise inputs for mathematical modelling, the creation of biosensors, and other applications in plant biotechnologies.

Mass spectrometry imaging and living single-cell mass spectrometry analysis could soon provide powerful tools for studying hormone distribution, even though they are still limited for hormone profiling [8]. Cell-specific sorting has been employed to gain more accurate insight into auxin [33,185] and CK [186] distributions in *Arabidopsis* root tips, and flow cytometric techniques for the sorting of organelles may soon provide a better view on subcellular distributions. Another possible approach for visualizing auxin and CK distributions at the cellular or subcellular level is using novel synthetic analogues labelled with 7-nitro-2,1,3-benzoxadiazole (NBD), for example, that have been recently developed to mimic native phytohormones in vivo [187,188,189]. A combination of all these methodologies with the use of mathematical modelling [78,122,190] to parameterize auxin homeostasis at cellular and subcellular levels will undoubtedly lead to far more detailed insights into the secrets of plant developmental control.

## Figures and Tables

**Table 1:** Auxin and cytokinin (CK) profiles at the subcellular level. Compounds are ordered according to their abundance in particular organelles. Abbreviations of auxins and CKs are listed in Figure A1.

Organelles (Species ^1^)	Auxins	Cytokinins	Reference
Chloroplasts (*Nicotiana tabacum*)	*Precursors* (n.a. ^2^) *Active compounds* (IAA) *Metabolites* (n.a.)	*Sum of CK bases* (*B*) *Sum of CK ribosides* (*R)* *Sum of CK N-glucosides* (*NG*) *Sum of CK O-glucosides* (*OG*) *Sum of CK phosphates* (*P*)	[29]
Chloroplasts (*Nicotiana tabacum, Triticum aestivum*)	n.a.	*B* (iP, DHZ) *R* (ZR, iPR, DHZR) *NG* (Z9G, DHZ9G, iPNG, Z7G, DHZ7G) *OG* (n.a.) *P* (iPRMP, ZRMP, DHZRMP)	[28]
Vacuoles (*Arabidopsis)*	*Precursors* (Trp, IAN, ANT, TRA, IAM) *Active compounds* (IAA) *Metabolites* (IAA-Glc, oxIAA)	n.a.	[30]
Vacuoles (*Arabidopsis, Hordeum vulgare*)	n.a.	*B* (*t*Z, iP) *R* (*c*ZR, iPR, *t*ZR) *NG* (iP7G, *t*Z7G, DHZ7G, *t*Z9G, iP9G, *c*Z9G, DHZ9G) *OG* (*c*ZROG, *c*ZOG, *t*ZOG, DHZOG, *t*ZROG) *P* (iPRMP, *t*ZRMP)	[31]

^1^ Phytohormone profiles are shown for species in bold. ^2^ “n.a.” indicates that the phytohormones were not profiled in the study.
